# Real-Time Protein and Cell Binding Measurements on Hydroxyapatite Coatings

**DOI:** 10.3390/jfb7030023

**Published:** 2016-08-27

**Authors:** A. M. Vilardell, N. Cinca, A. Jokinen, N. Garcia-Giralt, S. Dosta, I. G. Cano, J. M. Guilemany

**Affiliations:** 1 Centre de Projecció Tèrmica (CPT), Department Ciència dels Materials i Enginyeria Metal lúrgica, Universitat de Barcelona Martí i Franquès 1, Barcelona E-08028, Spain; sdosta@cptub.eu (S.D.); igcano@cptub.eu (I.G.C.); jmguilemany@cptub.eu (J.M.G.); 2 BioNavis Ltd., Hermiankatu 6-8H, 33720 Tampere , Finland; annika.jokinen@bionavis.com; 3 URFOA, IMIM (Institut Hospital del Mar d’Investigacions Mèdiques), RETICEF, Doctor Aiguader 80, Barcelona 08003, Spain; ngarcia@imim.es

**Keywords:** hydroxyapatite coating, multi-parametric surface plasmon resonance, biosensor, cell adsorption

## Abstract

Although a lot of in vitro and in vivo assays have been performed during the last few decades years for hydroxyapatite bioactive coatings, there is a lack of exploitation of real-time in vitro interaction measurements. In the present work, real-time interactions for a plasma sprayed hydroxyapatite coating were measured by a Multi-Parametric Surface Plasmon Resonance (MP-SPR), and the results were compared with standard traditional cell viability in vitro assays. MP-SPR is proven to be suitable not only for measurement of molecule–molecule interactions but also molecule–material interaction measurements and cell interaction. Although SPR is extensively utilized in interaction studies, recent research of protein or cell adsorption on hydroxyapatite coatings for prostheses applications was not found. The as-sprayed hydroxyapatite coating resulted in 62.4% of crystalline phase and an average thickness of 24 ± 6 μm. The MP-SPR was used to measure lysozyme protein and human mesenchymal stem cells interaction to the hydroxyapatite coating. A comparison between the standard gold sensor and Hydroxyapatite (HA)-plasma coated sensor denoted a clearly favourable cell attachment on HA coated sensor as a significantly higher signal of cell binding was detected. Moreover, traditional cell viability and proliferation tests showed increased activity with culture time indicating that cells were proliferating on HA coating. Cells show homogeneous distribution and proliferation along the HA surface between one and seven days with no significant mortality. Cells were flattened and spread on rough surfaces from the first day, with increasing cytoplasmatic extensions during the culture time.

## 1. Introduction

Hydroxyapatite (HA) coatings have been used during the last few decades surface orthopedic prostheses to enhance osseointegration and promote rapid bond fixation. Along with in vivo studies, in vitro experiments using different cells have revealed an enhancement in cellular adhesion, proliferation and differentiation to promote bone regeneration, although the mechanism of osteogenesis in response to HA coatings in in vivo tests still remains unclear [1,2,3,4,5,6,7].

After implantation in the human body, implant surfaces are immediately in contact with physiological fluids that contain lot of proteins that guide the adhesion of particular cell types to the surface. The adhesion of proteins, such as fibronectin, vitronectin, fibrinogen and collagen, is recommended to modulate cellular responses via integrin–ligand interactions and thus influence the subsequent cellular adhesion, proliferation and differentiation [8,9]. It is of interest to understand protein adsorption to solid surfaces, and it is a desirable characteristic on prosthesis because protein adsorption can trigger the adhesion of particles, bacteria or cells affecting the efficiency, longevity or the proper functioning of these medical devices.

Established in vitro, cell-based assays such as MTS (3-(4,5-dimethylthiazol-2-yl)-5-(3-carboxymethoxyphenyl)-2-(4-sulfophenyl)-2H-tetrazolium), BrdU (Bromodeoxyuridine) or ALP (Alkaline Phosphatase) assays in static cultures measure real-time interactions on the cellular level. These procedures are often based on labelled materials for imaging or detection purposes, where the final quantification is based on UV- or fluorescence spectroscopy, mass spectrometry, radiometry or chromatographic techniques. Thus, new in vitro cell-based assay methodologies and approaches, which enable direct detection, and real-time, non-invasive, label free and continuous high sensitivity monitoring of cell responses to stimuli, would be desirable.

### 1.1. Atmospheric Plasma Spray

Several techniques such as dip coating [10], sol-gel [11], electrophoretic deposition [12] and pulsed laser deposition [13] have been able to produce HA coatings, but Plasma Spray (PS) has been the most successful one from a commercial point of view and is the only one accomplishing the Food and Drug Administration (FDA) requirements [14].

PS is a thermal spray coating technology where the feedstock material is introduced into a plasma jet, emanating from a plasma torch at temperatures ranging from about 6000 to 15,000 °C, therefore high above the melting point of known materials. The plasma is generated by superheating an inert gas (argon or argon/hydrogen mixture). The feedstock material, normally powder, is injected into the gun via a carrier gas and it is propelled towards a substrate where the impacting droplets rapidly solidify and form a coating [15]. PS appears to be the most favorable mainly because of the high deposition rates at low cost. Nevertheless, the high temperatures from the PS technique lead to HA decomposition into many different secondary calcium phosphate phases with high dissolution rates in body fluids [16] such as tricalcium phosphate (α/β form), calcium oxide (CaO), tetracalcium phosphate (TTCP), along with amorphous calcium phosphate (ACP), increase dissolution rates. In addition, high temperatures do not allow the deposition of dopants. The ideal HA coating for orthopaedic implants would be one with strong cohesive strength, good adhesion to the substrate and low porosity [17], while few works attempt to report a preferred value of crystallinity [16]. Moreover, rapid cooling rates lead to HA microcracking questioning the fiability of the coating. Other methods to deposit HA are being studied as the denominated soft coatings e.g., HA coatings obtained by sputtering in order to avoid PS drawbacks [16].

Different biological static assays are found in the literature for plasma sprayed HA coatings, but none of them approach real conditions like dynamic assays do. Under dynamic culture conditions, cells attached faster onto HA surfaces. Dynamic cell culture was performed inside the Minucells^®^ (MINUCELLS and MINUTISSUE, Bad Abbach, Germany) flow perfusion. The system consists of a chamber, supplied by medium by a peristaltic pump at a 2 mL/h flow rate. The medium flows through the ceramic samples vertically in a bottom–up direction. A higher activity of alkaline phosphatase and larger number of grown cells were found in dynamic culture compared with static culture conditions [18]. Furthermore, dynamic conditions affect the formation of HA coating. The resulting microstructure (i.e., crystallinity, amount of phases with different solubilities) of the HA coating will also perform differently under static rather than dynamic conditions. Under dynamic conditions, the HA formation decreases with the increase of the flow rate, whereas under static conditions, it grows faster [19]. In addition, in vitro studies in dynamic conditions are more representative of what occurs in vivo than static conditions.

### 1.2. Multi-Parameter Surface Plasmon Resonance Technology

Among different surface detection methods (optical, mechanical, electrochemical), the optical ones are the most surface-sensitive label-free methods [20]. Surface Plasmon Resonance (SPR) is an optical detection method that has been used for a few decades for biomolecular interaction studies, like drugs and proteins [21]. SPR has reached popularity due to its high sensitivity and real-time measurements, which enables determination, not only of affinity, but also kinetics of the molecular interactions. Other techniques used in biosensors are QCM-D (quartz crystal microbalance with dissipation monitoring) and SAW (Surface Acoustic Wave).

SPR is based on an optical interfacial property, called the evanescent field, created into a dielectric interface under total internal reflection conditions. For measurement proposes, the evanescent field is enhanced with free electron excitation on a dielectric–metal interface. The evanescent field detects the optical density or a change in it, inside the evanescent field, which extends a wavelength (λ) of the incident light approximately to the measurement medium. The basic applications are the determination of kinetics and binding affinity between molecule interactions [20]. On the other hand, the QCM is a resonating device where the wave propagates in the bulk of the crystal at a typical operating frequency below approximately 20 MHz. Since the resonant frequency of the QCM device is altered by the adsorption of a thin layer of material on the device’s surface, this device can be used as a mass sensor. However, since the resonant frequency is dependent on the thickness of the piezoelectric material, there is a maximum operating frequency for any QCM device. Moreover, QCM-D is less ideal as an SPR method to quantify the amount of adsorbed serum proteins because it does not have the lowest detection limit. Furthermore, it is always challenging to accurately account for the water contribution to the signal [22]; SAW allows for an in-depth analysis of molecular interactions in real-time. Binding kinetics can be precisely determined by detecting mass and binding-induced conformational changes. The SAW technology measures changes in mass and conformation separately, thus providing new insights to mechanisms of binding in addition to the binding kinetics and stoichiometry. It is not only an excellent analytical tool for the detection of specific molecular interactions at solid–liquid interfaces, but also very popular due to its ability to operate in liquid media. It can also analyze interactions with whole cells, liposomes, membrane layers and whole viruses [23].

**Figure 1:**
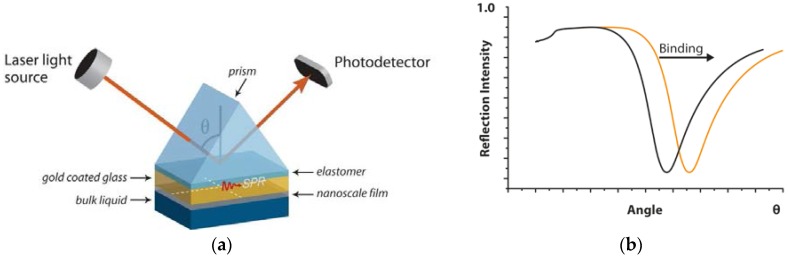
(**a**) measurements are performed on a sensor slide, typically a gold-coated glass slide that is placed between the prism and flow-cell. Multi-Parametric Surface Plasmon Resonance (MP-SPR) consists of an incident beam of *p*-polarized light that strikes electrically, and conducting sensors slide at the interface with high refractive index and an external medium (gas or liquid) with low refractive index. The incident beam jet moves at different angles and the determinate angle where surface plasmons exits take place, resulting in a reduced intensity of the reflected light and indicating changes in SPR signal due to surface molecular interactions; (**b**) the graph shows a shift in SPR due to formation of a layer at the surface. The *x*-axis is the angle at which the laser excites plasmons. The *y*-axis shows the level of light intensity reflected from the surface. The dip in the curve (lowest light intensity) shows when the plasmons are excited.

Physical SPR phenomena are not limited to molecular interactions but are also sensitive to other surface changes and applicable in the biomedical field [24,25,26]. In Multi-Parametric Surface Plasmon Resonance (MP-SPR) instruments, such as MP-SPR Navi™ 200 (BioNavis Ltd., Tampere, Finland), detection is based on the SPR principle utilizing the Kretschmann configuration and angle scanning optical arrangement (Figure 1). The SPR curve is detected when surface plasmons form on the metal surface that let a drop in the reflected intensity (Figure 1). The position of the SPR curve depends on the wavelength of the light, the refractive index on the measured surface and measurement media, such as air or water. MP-SPR measures full SPR curves on a wide angle range (laser rotates 38 degrees) and with two wavelengths of light (such as 670 nm and 980 nm). When binding on the surface is occurring, an SPR curve shift is detected due to the change in the refractive index near the surface. When the coating thickness increases up to hundreds of nanometers, the first SPR curve goes out of the measured angle range. However, two wavelengths used for measurement increase flexibility related to coating thicknesses due to the second SPR curve still in the measured angle range. The measurement of the wide angle range of MP-SPR also enables the measurement in a so-called waveguide mode, which can be utilized in SPR sensing when the thickness of the sample layer is >λ^−1^ of the incident light. The optical setup of MP-SPR Navi™ (BioNavis Ltd., Tampere, Finland) instruments is explained in details elsewhere [27,28]. The MP-SPR optical setup makes the method suitable for a wide range of coating materials such as hydroxyapatite. The MP-SPR instrument is proved to be suitable for the molecule–material interaction measurements and cell interaction studies such as a protein or block copolymers adsorption on a nanocellulose surface, a drug interaction with living cell monolayer and a cell binding to peptide functionalized surfaces [29,30,31,32]. SPR is extensively utilized in interaction studies; however, recent research of protein or cell adsorption on hydroxyapatite coatings for prostheses applications was not found. Different protein adsorbtion research was found for SAW and QCM technique on HA coatings. SAW studies also tested protein adsorption on electrophoretically deposited hydroxyapatite coatings and magnetron sputtered metallic with successful results [33]. QCM-D measurements on ~20 nm HA coatings show that the protein films adsorbed on HA had different viscoelastic properties depending on the HA crystal size [34] and clearly distinguish between different structures of adsorbed layers [35]. The correlation between protein adsorption (osteopontin) on HA surface and cell response was also evaluated. It was found that the amount of attached protein is related to a larger and faster cell spreading as well as higher cell motility [36]. However, no real-time cell measurements were found on HA coating.

## 2. Results and Discussion

### 2.1. HA Coatings by PS

**Figure 2:**
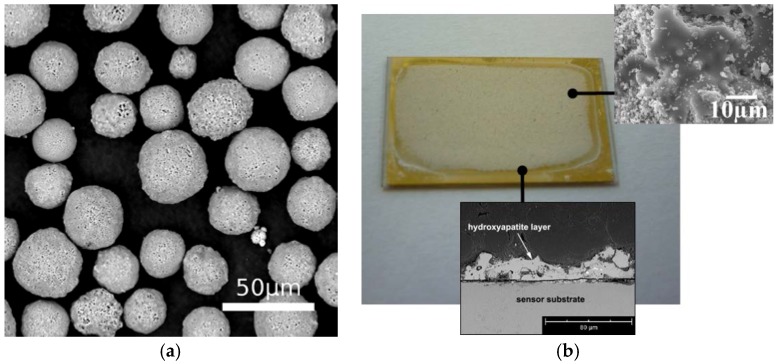
(**a**) SEM micrograph of hydroxyapatite (HA) particles and (**b**) free superface and cross area section of the HA coated sensor slide.

**Figure 3:**
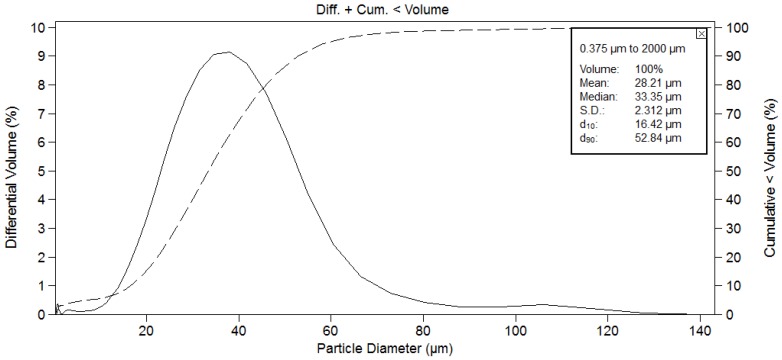
Particle size distribution of HA powder.

Figure 2a shows the morphology of the HA powder. It is a sintered powder consisting of spherical particles with a mean particle size of 28.2 μm and with a grain structure consisting of small equiaxed grains with an average grain-size of 0.5–2 µm. Particle size distribution is shown in Figure 3. The mean particle size is 28.2 μm, with Ø10 = 16.4 µm and Ø90 = 52.8 µm. The particles were sprayed onto TiO_2_ slide sensors following the spraying conditions mentioned in previous studies and specified in Table 1 [37]; they were optimized with the aim to achieve a higher crystallinity. Figure 2b shows a macroscopic view of the coated sensor as well as the cross section of the PS coating with an average thickness of 24.3 ± 5.7 µm measured by optical microscopy.

**Figure 4:**
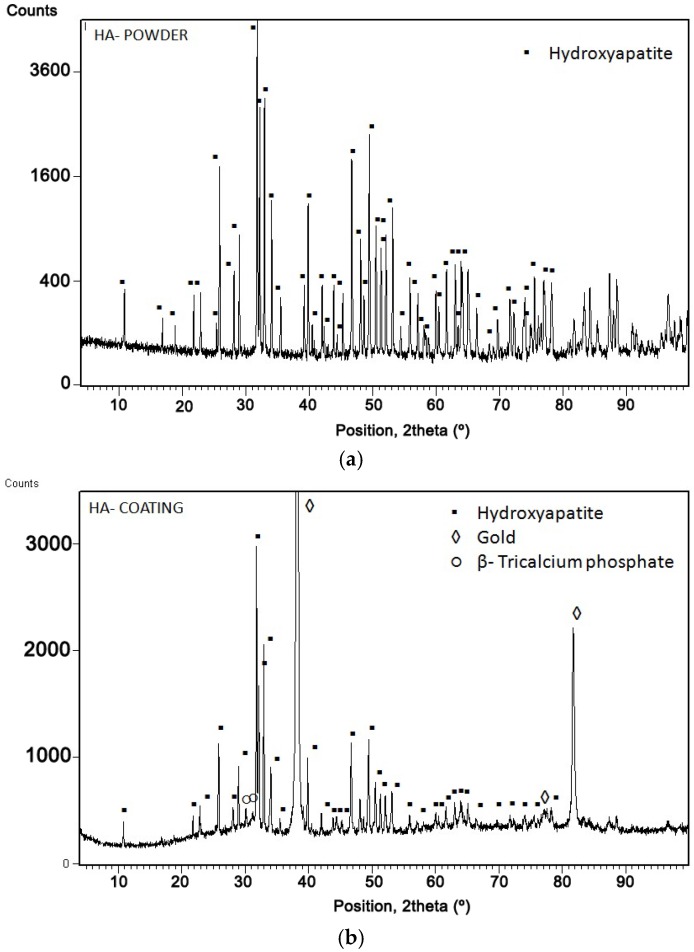
X-ray diffraction of (**a**) HA powder and (**b**) HA coating.

Figure 4 shows the X-ray diffraction of the HA powder (Figure 4a) and HA coating (Figure 4b). In both, the HA phase has been well identified according to the 01-089-6439 pattern of the JCPDS database. The XRD of the powder shows very narrow peaks indicating the high crystallinity of the feedstock powder, while the XRD of the HA coating on the sensor shows a non-smooth background indicating certain degree of HA amorphization. Moreover, apart from the intense gold peaks coming from the slide sensor material, the diffractogram shows certain lines belonging to β-tricalcium phosphate (β-TCP) due to HA decomposition at high temperatures.

It could be observed that the intensity of HA peaks decreased and an amorphous hump appeared in the coating (Figure 4b) in comparison with the powder (Figure 4a). This is due to the high temperature of the plasma flame, which results in full or partial particle melting. The melt could either (i) solidify to amorphous phases; (ii) recrystallize; or (iii) descompose to secondary. The fraction of the final phases was calculated according to Rietveld’s method and values of 61.78% for HA and 0.58% for β-TCP were obtained, as well as 37.64% of amorphous phase. The content of such phases strongly depends on the spraying conditions. The amorphous phases have high dissolution rates into body fluids, promoting faster osseointegration due to the release of Ca and P ions; however, it has been found that they have higher tendency to form at the coating-metal interface, which might be detrimental in the case that they dissolve before proper bone fixation [16]. In addition, the melt may be dehydroxylated and become oxy-apatite (OHA). The dehydroxylation creates a barrier to the nucleation of the HA or OHA from the melt and promotes the formation of the amorphous phase. The phase formation upon the droplet deposition will depend on (i) both the hydroxyl state and the cooling rate of the droplet during the solidification process; and (ii) the heat/hydroxyl accumulation during coating build up. HA coatings normally display an internal gradient structure from an amorphous base to a crystalline surface. During the spray process, the cooling rates of the first particles are controlled by rapid heat dissipation to the metallic substrate. With the coating build up, the cooling rate becomes smaller because the thermal conductivity of HA is much lower than of metals [38].

Previous in vitro tests with these HA-PS coatings as well as those in the literature, show that surface chemistry and topography of lower crystalline coatings could be favourable to cell attachment. However, cell proliferation reaches higher values with higher HA crystallinity [39]. In vivo studies reveal that high crystallinity coatings showed the higher shear strength and remained integrated with a bond, whereas the separation of the coating fragments is present in coatings having low crystallinity [40]. Thus, for maximizing crystallinity and minimizing impurity phases, a heat treatment is suggested [41], although not performed here.

### 2.2. Static Testing: Cell Viability and Morphology

HA coatings are known to dissolve in bone tissue and thus facilitate bone in-growth. However, there are contradictory views concerning this. Several authors stated that the surface degradation of calcium phosphate-based biomaterials seems to be closely related to osteoconductive properties [42,43] and plays an important role in initial implant fixation [42]. Bagambisa et al. [43] stated that extensive degradation/recrystallization events lead to the wide bone-bonding area. Maxian et al. [42] reported that restorable coating showed initial enhanced osteoconduction and comparable bone-attachment strength to non-resorbable coating in vivo. However, if only the osteoconductive property is desired for initial fixation, ACP coating may be advantageous, but if it is longevity that is desired, then crystalline HA coating is preferable [44].

**Figure 5:**
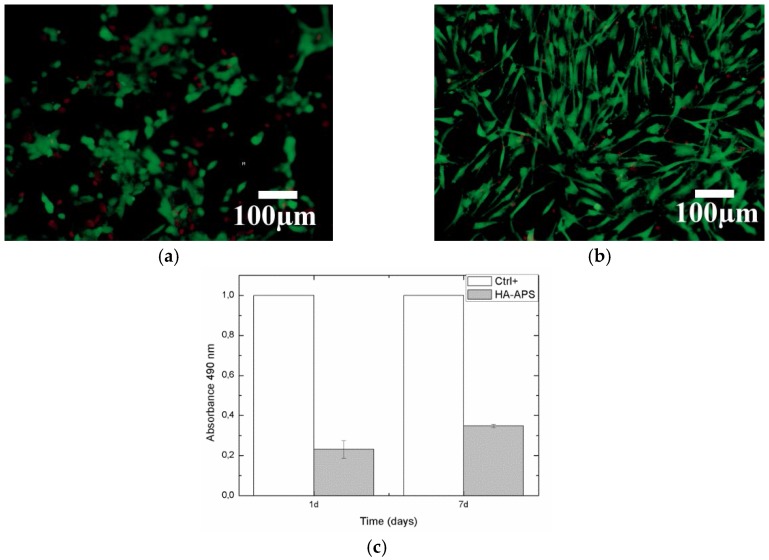
Live/Dead assay at (**a**) one and (**b**) seven days of human osteoblast culture onto HA coating; (**c**) human osteoblast cells proliferation measured by 3-(4,5-dimethylthiazol-2-yl)-5-(3-carboxymethoxyphenyl)-2-(4-sulfophenyl)-2H-tetrazolium (MTS) assay at one and seven days of culture onto HA coating (*n* = 3).

In our case, Live/Dead tests of human osteoblast (hOB) are shown in Figure 5a,b. The cell number on the surface increases exponentially with time. The distribution is homogeneous all across the surface, and osteoblast mortality is low from one to seven days. A 61.78% of crystalline HA phase will help coating fixation, but amorphous and β-tricalciumphospate will help to enhance osseointegration [45]. hOB proliferation was measured by MTS activity in Figure 5c. MTS activity increased with culture time indicating that cells were proliferating on HA coating.

In general terms, many authors have observed that the higher the crystalline content, the better the cellular response [46,47,48]. For example, Yang et al. [46] obtained a lower crystallinity value (17.7% of amorphous phase content) [46] and observed that an hydrothermal treatment could be also used to improve the mechanical strength, crystallinity (9.1% of amorphous phase) and phase composition of HA for long-term mechanical and biological fixation of the implant. Other alternatives such as thermal treatment were performed in order to enhance crystallinity [47], also leading to an improved corrosion resistance due to a coating surface modification with higher crystallinity and less dissoluble nonapatite phases (TCP), as well as a reduction of coating defects when plasma-sprayed coatings were subjected to postdeposition heat treatment. Furthermore, the use of HA composite powders such as carbon nanotube (CNT)-reinforced HA enhanced crystallinity (by 27%) and fracture toughness (by 56%) when compared with that of plasma-sprayed HA without CNT reinforcement [48].

**Figure 6:**
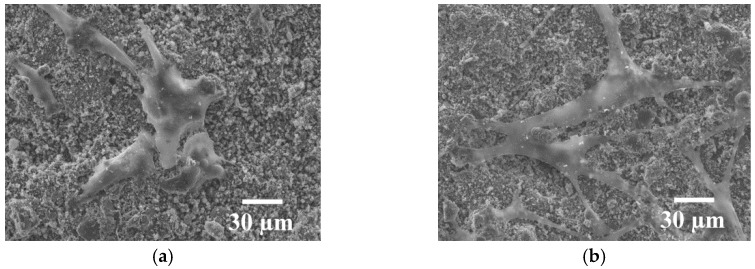
FESEM micrographs of human osteoblast cells at (**a**) one and (**b**) seven days of culture onto HA coating.

FESEM observation provides a detailed look at the morphology of the adhered cells (Figure 6a,b). Cells were able to attach and spread on HA coating. Cells have an osteoblastic shape that is maintained within the days with filopodia extending over them. At one day, cells have a more round-shape because they start being attached on the surface. At seven days, cells start acquiring spindle-shaped morphology, anchored, attached, spread and proliferated on the HA surface. Neighboring cells maintained physical contact through multiple extensions.

### 2.3. Dynamic Testing

#### 2.3.1. MP-SPR Protein Measurement

**Figure 7:**
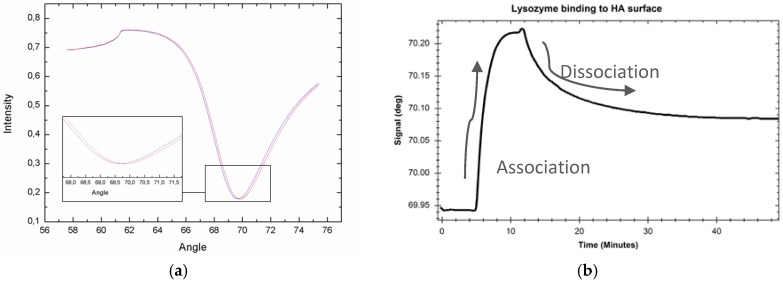
(**a**) Full SPR curve before lysozyme protein injection (blue curve) and after protein deposition (red curve) on an HA coating; (**b**) sensogram showing SPR Peak Minimum Angle changes during Lysozyme interaction.

Lysozyme binding to HA coating was measured with the MP-SPR (Figure 7). Figure 7a shows SPR curve before and after lysozyme injection where curve displacement to the right is observed due to protein binding. Figure 7b shows centroid sensogram during interaction measurement; it presents changes in SPR peak minimum position during binding. Lysozyme injection is started at 7.5 min, and, due to the advantage of real-time measurement, it is observed that lysozyme binds readily on the HA coating (association curve) with used concentration reaching plateau a couple minutes after injection (Figure 7b). Since injection is ended at 14.5 min, protein starts dissociating from the surface and reaches a plateau after approximately 20 min dissociation (time point 34.5 min).

Regarding the previous mentioned techniques, it was also proved the applicatiblity of Physical Vapor Deposition (PVD) and Electrophoeric deposition (EPD) time for SAW biosensor functionalization. Moreover, it was demonstrated that protein adsorption such as fibronectin can be detected on these metallic and ceramic (HA) surfaces [33]. In addition, other studies indicate that the HA coated sensor by EPD is applicable for qualitative and conformational analysis of protein adsorption with QCM-D techniques [35]. Studies were performed in order to study the effect of crystallite size, and it was found that the adsorption amount of human serum albumin was affected by crystal size but not that of bovine plasma fibrinogen [34].

#### 2.3.2. MP-SPR Cell Measurement

**Figure 8:**
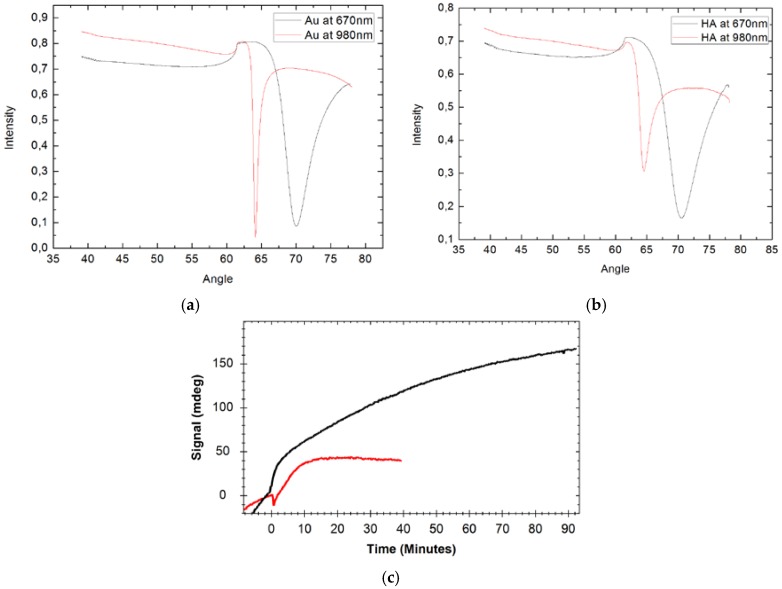
Full SPR curves measured in cell culture medium of (**a**) pure gold and (**b**) HA coated sensor slide; and (**c**) measured peak minimum angle sensograms during Human mesenchymal stem cells adsorption on the HA coating (black) and the gold (red) surface. Measured with 670 nm wavelength.

Human mesenchymal stem cells derived from adipose tissue (AD-MSC) adsorption on the HA coating and reference gold surface were measured using MP-SPR. Full SPR curve in cell culture medium at wavelengths of 670 and 980 nm was measured before cell attachment on the gold sensor slide (Figure 8a) and HA surface on a TiO_2_ coated gold sensor slide (Figure 8b). HA coated surface was compared to the gold surface (Figure 8c). HA surface shows faster increase of signals than gold surface indicating faster attachment of the cells as seen during the first minutes of the cells injection (from time point of 0) (Figure 8c). Cells attach over time on the HA surface until reaching plateau value at 90 min, whereas on a gold surface, the plateau value is reached only after 10 min. Despite that, AD-MSC cells have a good tendency to attach easily on surfaces; results show that HA surface favours attachment of the cells compared to the gold surface. The HA is porous material, and it is expected that HA coatings have more binding capacity on the surface compared to a smooth gold surface. However, higher porosity is not solely explaining detected differences of the cells attachment.

## 3. Experimental Section

### 3.1. HA Coatings by PS

Commercial Captal 30 HA powder from Plasma-Biotal Ltd. (Tideswell, UK) was used as feedstock. Typical particles were analyzed with Scanning Electron Microscopy (SEM) ProX Phenom (Eindhoven, the Netherlands). Particle size distributions were measured by using a Laser Diffraction Particle Size Analyser Beckman Coulter LS 13320 (Brea, CA, USA). XRD patterns of powder and coating of HA were obtained by a PANalytical X’Pert PRO MPD θ/θ Bragg-Brentano powder diffractometer (Madrid, Spain) of 240 millimetres of a radius that uses Cu-Kα radiations (α = 1.5418 Å) at 45 kV, 40 mA, and a θ/2θ scan from 5 to 100° 2θ with step size of 0.017° and measuring time of 100 s per step. A Rietveld analysis, using the FullProf software (FullProf_Suite version, CEA-CNRS, France) [49], was carried out to refine the lattice parameters, determine the crystallite size and to find the percentage of the crystalline phases and amorphous phase [50]. HA particles were sprayed onto TiO_2_ sensor slides provided by Bionavis Ltd. (Tampere, Finland) using an PS A-3000S torch (Sulzer Metco, Wohlen, Switzerland).

### 3.2. MP-SPR Protein and Cell Measurements

Binding of Lysozyme protein and human mesenchymal stem cells derived from adipose tissue (AD-MSC) were measured onto HA coating which was deposited on a TiO_2_ coated gold sensor slide, and as reference onto an uncoated gold sensor slide (for AD-MSC). Measurements were performed with MP-SPR Navi™ 200 instrument (BioNavis Ltd., Tampere, Finland) in angular scan measurement mode and at temperatures of 20 °C and 21 °C, respectively. For lysozyme (0.2 mg/mL) interaction, a running buffer was 0.15 M NaCL, flow rate 30 μL/min and measurement was done with 670 nm laser wavelength.

For AD-MSC cells, those were cultivated and provided by the Adult Stem Cell Group, BioMediTech (University of Tampere, Tampere, Finland) and grown according standard procedures. The cells were washed with PBS (Phosphate Buffered Saline) and the medium was replaced and dispersed in a cultivation bottle by a cell scraper, resulting in approximately 0.1 million cells per mL concentration in cell culture medium. MP-SPR measurements were done simultaneously with 670 and 980 nm wavelengths using a steady-state cuvette. AD-MSC suspension was injected to the cuvette and the interaction was monitored until the cell attachment seemed to reach a plateau value. The results of AD-MSC attachment were compared between HA coated TiO_2_ slide sensor with gold standard sensor slide.

Measured MP-SPR data combined with earlier published results indicates that MP-SPR is suitable method for hyrdoxyapatite coating interaction studies, however, repetitions would be needed to determine reproducibility of the results.

### 3.3. Cell Viability and Morphology

Human osteoblastic cells (hOB) have been obtained from knee trabecular bone after prosthesis replacement following the protocol described by Nacher et al. [51]. The entire study has been conducted approved by the of Parc de Salut Mar Ethics Committee (Barcelona, Spain). Finally, cells were passaged into new 75 cm^2^ flasks until the needed number was reached. A maximum of a third subculture has been used in the experiments. For material testing, samples were sterilizated overnight in ethanol 70°, washed in PBS and placed on a 48-well polystyrene culture plate (Nunc A/S, Barcelona, Spain). Each sample was seeded with 100,000 cells and cultured with Dulbecco’s Modified Eagle Medium (DMEM) supplemented with 10% FBS and ascorbic acid.

A LIVE/DEAD Viability/Cytotoxicity Kit for Mammalian Cells (Invitrogen, Carlsbad, CA, USA) was used for characterized cell viability, attachment and distribution at one and seven days of cell culture. Live cells can be easily distinguished from dead cells by simultaneously staining them with green-fluorescent calcein-AM (acetoxymethyl) to indicate intracellular esterase activity (live cells) and red-fluorecent ethidium homodimer-1 to indicate loss of plasma membrane integrity (dead cells). The surfaces have been then observed with a Leica DM 1000 optical microscope (Barcelona, Spain). Cell proliferation has been tested at one and seven days of culture using the MTS assay CellTiter 96^®^ AQueous One Solution Cell Proliferation assay (Promega, Madrid, Spain) according to the manufacturer’s protocol. Seeded materials were changed to a new empty well in order to assay only the cells adhered to the material. Then, 50 μL of MTS were added in each sample cultured with 250 μL of supplemented medium, incubated for 3 h and then the absorbance at 490 nm was recorded. Results were normalized by control well (without material) within each experiment.

Cell morphology has been determined by fixing cells on the coating surface with 2.5% gluteraldehyde in PBS buffer 1 h at room temperature at one and seven days of culture. Then, a progressive dehydratation with alcohol and critical point drying was performed. The samples have been observed using a Jeol JSM-7100F FESEM (Peabody, MA, USA) at 15 kV, after being sputtered to turn them conductive.

All test cell viability and morphology test were performed three times in order to ensure their reproducibility.

## 4. Conclusions

Plasma Spray hydroxyapatite coatings were successfully produced onto TiO_2_ sensor slides with good adherence, and the protein and cell attachment onto such HA coatings, measured using the MP-SPR method, indicated an increase in protein and cell binding in comparison with the non-coated sensor slide, meaning an enhancement of bond osseointegration for prosthesis applications. The results of this work demonstrate the feasibility of using the MP-SPR process to evaluate real-time in vitro interaction measurements and open a new route to assess the performance of bioactive surfaces at a further step than just by observing the cell viability and proliferation that most routine studies present.

## Figures and Tables

**Table 1:** Plasma spraying conditions.

Primary Gas (Ar), Flow Rate (L/min)	Secondary Gas (H), Flow Rate (L/min)	Powder Carrier Gas (Ar), Flow Rate (L/min)	Arc Current (A)	Stand-off Distance (mm)	Torch Speed (mm/s)
50	1	3.65	500	80	600
